# Optimization of dynamic incentive strategies for public transportation based on reinforcement learning and network synergy effect

**DOI:** 10.1038/s41598-025-16632-y

**Published:** 2025-08-21

**Authors:** Yifang Chen, Shunlin Wang

**Affiliations:** 1 Office of Academic Affairs, Ningbo Polytechnic University, Ningbo, 315800 China; 2 School of Supply-Chain Administration, Ningbo Polytechnic University, Ningbo, 315800 China

**Keywords:** Reinforcement learning, Network synergy, Public transportation, Dynamic incentives, Strategies, User heterogeneity, Environmental sciences, Engineering, Mathematics and computing, Applied mathematics, Computer science

## Abstract

Aiming at the challenges of peak passenger congestion, user behavior heterogeneity and insufficient network synergy faced by public transportation systems in urbanization, this study proposed the Dynamic Incentive Strategy-Heterogeneous Response Synergy Model (DIS-HARM). The model integrated reinforcement learning, user heterogeneity modeling and small-world network synergy mechanism, adjusted the carbon credit intensity in real time by dynamic incentive generator, quantified the diminishing marginal utility effect of incentives for high-income groups by combining elastic user identifiers, and designed weather attenuation coefficients to optimize the spread of social influence. Simulation results showed that DIS-HARM significantly improves system efficiency and fairness: the peak hour passenger flow reduction rate reaches 72.2% (2.5% higher than the static strategy), the average peak hourly cost is reduced by 3.125%, and 36.5% of the incentive resources are tilted to the low-income group (83.1% coverage rate) at the same time. The model provided a theoretical tool for dynamic pricing and differentiated incentive strategies for urban transportation management, helping to achieve the dual goals of green travel and social equity.

## Introduction

Urban public transportation systems are facing increasing challenges of traffic congestion, environmental degradation and uneven resource allocation. The efficient operation of public transport systems is crucial for sustainable urban development, especially under the pressure of peak hour traffic, how to optimize travel behavior through dynamic incentive strategies has become a hot research topic. Traditional static incentive mechanisms (e.g., carbon coin incentives and fixed pricing) are often difficult to adapt to dynamic demand fluctuations and heterogeneous user preferences, while existing research on dynamic incentive strategies focuses on economic incentives, information guidance, or carbon coin mechanisms, which are also deficient in user heterogeneity, multi-initiative synergies, and network effects. For example, although the carbon coin incentive model proposed by Zhou et al. [1] can alleviate peak congestion, its assumption of user homogeneity ignores the time sensitivity and income variability caused by differences in socio-economic backgrounds, and Xu et al. [2] share the traffic flow allocation through parking preferences, but do not consider the propagation effect of social influence in multi-object networks. These limitations highlight the need for adaptive strategies that need to incorporate real-time feedback, user diversity, and network synergies.

In recent years, advances in reinforcement learning (RL) and multi-subjective systems have provided new ideas for the above problems, e.g., Cortina et al. [3] proposed an equilibrium model for mixed strategies (e-tickets and road pricing) in public–private transportation networks through a dynamic user equilibrium model with multi-objective Bayesian optimization, which can effectively alleviate congestion and optimize the efficiency of the system, but its framework still fails to cover the spatial fairness and user behavior heterogeneity. Comprehensively, existing literature shows that there are still three major limitations in current research: (1) most models assume user homogeneity and ignore the impact of heterogeneous characteristics such as income and time sensitivity on incentive response; (2) strategy optimization has a single objective (e.g., focusing only on efficiency or environmental protection) and fails to synergize multidimensional objectives (e.g., fairness and user satisfaction); (3) most studies lack synergistic effects of user’s social network and group quantitative modeling, which is difficult to support the global optimization decision.

To address the above research gaps, this study proposes Dynamic Incentive Strategy-Heterogeneous Response Collaborative Model (DIS-HARM), which integrates a reinforcement learning framework, a user heterogeneity decision-making mechanism and a multi-subjective collaborative network, and the core innovations include (1) a dynamic incentive generator based on Q-learning. Dynamically adjusts the carbon coin incentive strength (0.5Y ~ 2 CNY) according to the real-time passenger density and adoption rate to optimize the diversion efficiency during peak hours. (2) Heterogeneous user decision model. Introduce the time-rigid parameter of Pareto-distributed income and Sigmoid mapping to portray the diminishing marginal utility effect of high-income groups on incentives. (3) Watts-Strogatz small-world network. Simulate the social influence propagation mechanism to shape individual choices through neighbor adoption states together with spatial fairness constraints.

The theoretical contribution of this study is to expand the intersection of dynamic incentive strategies and user heterogeneity modeling, which provides new ideas for multi-objective optimization of public transportation systems. On the practical level, the DIS-HARM model can provide dynamic pricing, differentiated incentive allocation and social communication strategy design tools for urban transportation management, helping to achieve the dual goals of green travel and social equity.

The structure of this paper is as follows: Chapter 2 reviews the research progress of dynamic incentive strategies, user heterogeneity and network effects; Chapter 3 details the DIS-HARM model architecture, including the state-action space design, utility function and network dynamics formulations; Chapter 4 compares the performances of DIS-HARM and static strategies through numerical simulation to analyze the efficiency, fairness and network synergistic effects; Chapters 5 and 6 respectively discuss the research significance, limitations and conclusions, etc.

## Literature review

### Research progress on dynamic incentive strategies

Dynamic incentive strategies, as an important means to optimize public transportation systems, have received extensive attention in recent years. Existing studies mainly regulate travel behavior through economic rewards, information guidance and carbon coin mechanism. For example, some scholars for the first time combined carbon coin incentives with MaaS platform, proposed a staggered travel guidance mechanism, quantified stochastic incentive effects through the additive/multiplicative criterion, and revealed the trade-off relationship between the budget and penetration rate. This study shows that dynamic incentives can significantly change commuters’ departure patterns, but it assumes user homogeneity and does not cover heterogeneous group behavioral differences [1]. Similarly, multi-modal traffic flow allocation is optimized by parking preference incentives, but the matching success formula may fail in extreme scenarios. Recent works integrate Bayesian optimization for public–private transport synergy [2].

In terms of technology-driven dynamic strategies, the proposed EV rebalancing framework based on Multi-intelligent Reinforcement Learning (MARL) is optimized to dynamically extend the system’s non-smooth action space through action cascading strategies. Its simulation results show that the model can improve the net gain by 20%, but the generalization ability is limited and the spatial fairness is not considered [3]. Recent MARL frameworks enhance dynamic adaptability in large-scale systems, such as fleet rebalancing for shared mobility [4] and multimodal collaboration benchmarks [5]. These address scalability challenges in peak-hour traffic management. In addition, some scholars use inverse Stackelberg game with deep reinforcement learning to design eco-driving incentives, but their experiments are based on small-scale simulation scenarios and do not validate real traffic complexity [6].

The introduction of reinforcement learning techniques further improves the adaptability of dynamic incentives. The two-level policy network (ac-PPO) proposed in the literature [4] handles the non-smooth action space of the dynamically scaling system, but fails to address the fairness issue [5]. Literature [7], on the other hand, reduces the incentive cost by a factor of 8 through an organization-level incentive framework, but assumes that driver route adjustments are fully mandatory, thus ignoring individual stochasticity [8].

### Challenges and breakthroughs in user heterogeneity modeling

User heterogeneity has a significant impact on the effectiveness of motivational strategies. Earlier studies mostly portrayed heterogeneity through categorical variables (e.g., income, age), while recent approaches focus more on multidimensional feature modeling. Field experiments quantify time-value heterogeneity [9], while advanced choice models capture psychological factors [10].

In terms of heterogeneity quantification methods, the integration of land-use characteristics and discrete choice models reveals that disadvantaged groups suffer higher welfare losses due to the increased number of interchanges, but it does not simulate the dynamic changes in road conditions after the implementation of the policy [11]. In addition, after the introduction of a taxonomy based on observable equity objectives, scholars have empirically demonstrated the “triple bottom line” benefits of personalized pricing in vaccine subsidies, but it relies on monotonic demand assumptions and fails to address the impact of unobserved behavioral biases [12].

Existing models still face both data and theoretical challenges. Literature [13] proposes a continuous-time model to address the time aggregation bias of the traditional BPP approach, but its capital gains assumption may introduce measurement error [14]. Scholars combined subjective and objective data to construct SEM models [15] for related studies, but the cross-sectional data cannot infer causality [16].

### Multi-agent collaboration and network effect

Personalized dynamic pricing leverages activity-based demand models [17] to optimize resource allocation. Researchers have proposed a maturity model for Collaborative Intelligent Transportation Systems (C-ITS) that defines key capabilities such as strategic policies, data sharing, etc., but its model validation relies on a limited sample of experts and does not cover technical infrastructure indicators [18]. Similarly, although a city maturity assessment framework was developed for collaborative public transportation systems, it did not address the issue of legal synergy across jurisdictions [19].

In terms of network effect modeling, personalized recommendation was achieved by fusing TF-IDF, K-means and Q-learning reinforcement learning, but its data time window was short and did not cover real-time dynamic factors [20]. Literature [21] firstly applied the theory of social network centrality to transit network assessment, but did not include metro data. And in literature [22], a two-layer planning model is constructed to optimize the multi-objective equilibrium by combining electronic road tickets and road tolls, but its assumption of monotonically differentiable travel time function of road sections restricts the practical application scenarios. Literature [23] uses generalized Colonel Blotto game theory to design an incentive mechanism to optimize the allocation of rewards from the server to the computing nodes, which improves the performance and convergence speed of the location-based federated learning model. However, its computational complexity grows polynomially with the number of nodes, leading to high computational cost and runtime.

In summary, there are three major limitations in existing studies: (1) Existing dynamic incentive studies mostly rely on static parameter assumptions (e.g., user homogeneity, fixed budgets), and lack real-time feedback and adaptive adjustment capabilities. At the same time, the optimization objectives of incentive strategies are single (e.g., focusing only on efficiency or environmental protection) and fail to synergize with multidimensional objectives (e.g., fairness, user satisfaction). (2) Existing models mostly adopt discrete grouping or linear parameterization methods, which are difficult to capture the complex nonlinear features of user decision-making (e.g., risk preference, social network influence). In addition, there is insufficient research on the synergistic mechanism of heterogeneity modeling and dynamic incentives, which makes it difficult to adapt the strategy to diverse needs. (3) Existing synergistic models mostly focus on a single subject or static network, and lack the design of multi-subject dynamic interaction mechanisms. At the same time, there are insufficient quantitative methods for network effects (e.g., travel behavior diffusion, information dissemination), which makes it difficult to support the realization of global optimization goals. Colonel Blotto models computational complexity grows polynomially with the number of nodes, and as dynamic nodes increment in dynamic traffic incentive scenarios, performance bottlenecks occur with the surge in computational complexity when processing large-scale data.

To comprehensively address the above three major limitations (static parameters, linear heterogeneity modeling, and static network effects), Table 1 synthesized all cited studies into four innovation dimensions. Literature was grouped by methodological focus (e.g., [1, 3, 4] for dynamic incentives; [7–10, 12] for user heterogeneity), enabling a direct visual contrast between prior approaches and DIS-HARM’s integrated breakthroughs. This holistic analysis demonstrated that DIS-HARM’s novelty lied not in component combination but in synergistic mechanisms (e.g., meta-learning rewards, ηᵢ elasticity) that resolved cross-dimensional gaps.

**Table 1:** Holistic innovation comparison across research dimensions.

Research dimension	Prior approach limitations (selected examples)	DIS-HARM innovation
Dynamic Adaptation	•Static carbon coins ([1]) •Fixed pricing ([2]) •Non-adaptive MARL ([3])	Meta-learning reward coefficients​ (Eq. 5): Real-time cₚ/cₒ updates
User Heterogeneity Modeling	• Linear VOT ([9]) • Discrete grouping ([10]) • Rigid probit models ([15])	​ηᵢ elasticity + Sigmoid βᵢ​ (Eq. 7,11): Nonlinear cross-modal response
Network Synergy	• Static maturity models ([18]) • TF-IDF recommendations ([20]) • Centrality-based assessment ([21])	Weather-modulated propagation​ (φ(wₜ) in Eq. 13): Dynamic influence decay
Multi-objective Optimization	• Efficiency-only focus ([3, 20]) • Unbalanced subsidies ([12])	​**Fairness-efficiency trade-off**​

## Materials and methods

**Fig. 1:**
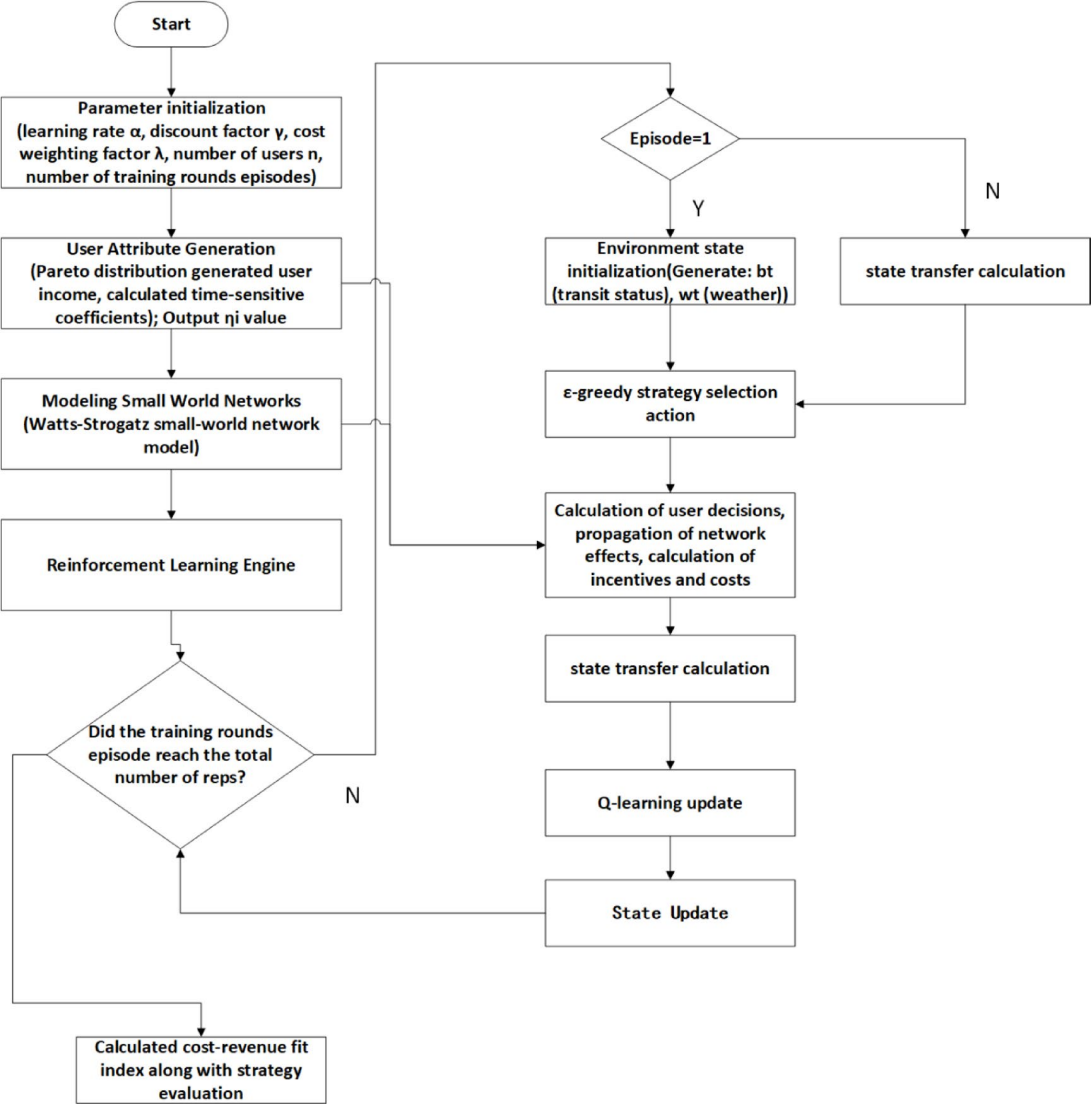
Framework of the modeling study.

Based on the research gaps revealed by the literature review, this study proposed the Dynamic Incentive Strategy-Heterogeneous Response Collaborative Model (DIS-HARM) , which integrated the reinforcement learning framework, heterogeneous user decision-making mechanism and multi-subject collaborative network, aiming at solving the key issues of insufficient adaptability of the static strategies, missing heterogeneous demand modeling, and imperfect group interaction mechanism in the existing researches. The architecture of the model was shown in Fig. 1, and the model design was detailed in the following three modules: dynamic incentive generator, heterogeneous user decision-making model, and multi-subject collaborative network construction. To address the key shortcomings of multi-modal synergy and dynamic connectivity, this study extended the DIS-HARM framework through two elements: (1) multi-modal synergy: the introduction of resilient users (${\eta }_{i}$) to characterize groups that were more responsive to incentives (transferable across transportation modes), and the inclusion of transit system state (${b}_{t}$) into the reward function to promote transit resource utilization during peak hours; and (2) dynamic connectivity: through the effects of weather on the decision threshold (δ) and social influence decay (φ) modulation to quantify the environmental impact on behavioral adoption and avoid topological complexity. These extensions were used to inject realistic complexity while maintaining computational tractability through localized tuning of the decision and propagation functions.

### Dynamic incentive generator, DIG

#### State-action space design

A dynamic strategy optimization framework was designed based on Q-learning with the aim of generating dynamic incentive signals through real-time data feedback to optimize the diversion efficiency for travelers traveling during peak hours.

Considering 24 h a day, public transportation faces certain peak and peak-off states every day. Traditional studies determined peak/peak-off states based on fixed time windows (e.g., 7:00–9:00 as peak). To address this limitation, we propose a dual-peak state management model integrating ​time period characteristics and dynamic diversion strategies. The model realized dynamic management through two core mechanisms of environment initialization (Mode 1) and state transfer (Mode 2), which were mathematically expressed as follows: first, environment state initialization (Mode 1). Define the discrete time series $t\in \left\{\text{1,2},\cdots ,T\right\}.$ Considering multimodal synergy and dynamic environmental impacts, the state of the environment was described by the quintuple $({s}_{t},{p}_{t},{d}_{t}, {b}_{t}, {w}_{t})$, where: ${s}_{t}\in \left[1, 2\right]$ was the state coding (1: peak state; 2: peak-off state); ${p}_{t}\in {\mathbb{R}}^{+}$ was the time period characteristic parameter; and ${d}_{t}\in \left[0, 1\right]$ was the area density parameter; ${b}_{t}\in \left[0, 1\right]$ represented the state of the transit system (0: extremely congested/unavailable; 1: idle and available); ${w}_{t}\in \left\{1, 2\right\}$ denoted the state of the weather (1: normal; 2: inclement), with $P({w}_{t}=1)=0.8$ and $P({w}_{t}=2)=0.2$, and this parameter quantified the effect of the external environment on the system’s connectivity.

The state initialization process was defined by the following set of Eqs. (1):

1 $$\left\{\begin{array}{c}h\left(t\right)=\left[\left(t-1\right)mod 24\right]+1 \\ {s}_{t}=\left\{\begin{array}{c}1, h(t)\in \left[7, 9\right]\cup \left[17, 19\right]\\ 2, otherwise\end{array}\right.\\ \begin{array}{c}{d}_{t}\sim \left\{\begin{array}{c}U\left(0.7, 0.9\right), {s}_{t}=1\\ U\left(0.3, 0.5\right), { s}_{t}=2\end{array}\right.\\ {b}_{t}\sim \left\{\begin{array}{c}U\left(0.3, 0.8\right), {s}_{t}=1\\ U\left(0.6, 0.9\right), { s}_{t}=2\end{array}\right.\end{array}\end{array}\right.$$

where $h\left(t\right)$ denoted the current time period (hour) and $U\left(a, b\right)$ was the uniform distribution on the interval $\left[a, b\right]$. This design realized the bimodal feature modeling of the morning peak (7:00–9:00) and the evening peak (17:00–19:00).

Second, the state transfer mechanism. Define the state transfer function $f: \mathcal{S}\times \left[0, 1\right]\to \mathcal{S}$ (implemented in Appendix A.1), and the formula was shown in Eq. (2).

2 $${s}_{t+1}=f\left({s}_{t},{r}_{t}\right)=\left\{\begin{array}{c}2, { r}_{t}\ge \tau \\ {s}_{t}, otherwise\end{array}\right.$$

where ${r}_{t}$ was the adoption rate at time $t$ and $\tau$ was the adoption rate threshold.

In order to encourage the travelers to travel green, the study considered to give the travelers a certain amount of carbon coins as the incentive value, drawing on the research of literature [1], from the basic compensation to the high incentive for four levels of intensity of the coverage, which formed the action space as $${a}_{t}=\left\{\begin{array}{ccc}0.5& 1.0& \begin{array}{cc}1.5& \left.2.0\right\}\end{array}\end{array}\right.$$ (unit: CNY).

#### Q-learning optimization mechanism

Dynamically adjusting the state-action value matrix (Q-table) according to the environmental state feedback, this study adopted the Bellman’s optimization equation to update the Q-value (code in Appendix A.4), see Eq. (3).

3 $$\begin{array}{*{20}c} {Q\left( {s_{t} ,a_{t} } \right) \leftarrow \left( {1 - \alpha } \right)Q\left( {s_{t} ,a_{t} } \right) + \alpha \left[ {R\left( {s_{t} ,a} \right) + \gamma max_{a} Q\left( {s_{t + 1} ,a} \right)} \right],} \\ \end{array}$$

where $\alpha$ was the learning rate; $\gamma$ was the discount factor, $R({s}_{t},a)$ was the reward at moment t, and ${s}_{t+1}$ was the next state.

In the Q-table initialization process, we first created a 2 × 4 matrix filled with zeros, representing 2 states and 4 possible actions in the system. Each entry stored the expected value (Q-value) of taking that action in that state. To break symmetry and improve exploration efficiency, we added small random perturbations generated by 0.01 * randn(2, 4), normally distributed values with zero mean and low standard deviation to the initial zero values. This ensured that different actions had slightly different starting values, preventing the agent from being indifferent among them.

In this implementation (See Appendix A.1.), we used the ε-greedy strategy to select actions, balancing exploration and exploitation. Specifically, the agent looked up the action values for the current state in the Q-table Q, and with probability epsilon, it selected a random action to explore; otherwise, it chose the action with the highest Q-value (i.e., the best-known action).

In order to realize time period differentiated incentives, the reward coefficient set in the peak time should be larger than the reward coefficient in the flat time period, and at the same time, the intensity of the punishment for the users who did not adopt was adjusted by $\lambda$ to avoid overspending, In order to incentivize the full use of transit resources during peak hours. The incentive function was extended to include peak hour transit synergy benefits.

Adaptation was achieved through meta-learning (online gradient updating), dynamic coefficients were adjusted according to the extent to which the average adoption rate $\overline{r }$ deviated from the target $\tau$ , and the clip function ensured that the coefficients were within a reasonable range to avoid over-adjustment.

and the corresponding reward function was shown in Eq. (4).

4 $$R\left({s}_{t},a\right)=\left\{\begin{array}{c}{c}_{p}\bullet AdoptRate+k\bullet {b}_{t}\bullet AdoptRate-\lambda \left(1-AdoptRate\right), {s}_{t}=1 \\ {c}_{o}\bullet AdoptRate-\lambda \left(1-AdoptRate\right), {s}_{t}=2\end{array}\right.$$

where k was the coefficient of synergistic benefits, and in this study it was assumed that k = 0.5.$\lambda$ was the cost weight coefficient, $AdoptRate$ was the adoption rate (the proportion of users responding to the incentive), ${c}_{p}$ and ${c}_{o}$ were dynamic coefficients updated every K episodes. See Eq. (5).

5 $$\begin{array}{*{20}c} {\begin{array}{*{20}c} {c_{p} \leftarrow clip\left( {c_{p} + \zeta \cdot \left( {\tau - \overline{r}} \right),c_{p}^{min} ,c_{p}^{max} } \right)} \\ {c_{o} \leftarrow clip\left( {c_{o} + \zeta \cdot \left( {\tau - \overline{r}} \right),c_{o}^{min} ,c_{o}^{max} } \right)} \\ \end{array} , } \\ \end{array}$$

With $\zeta =0.1$(learning rate), $\tau =0.7$(target adoption rate), $K=10$, ${c}_{p}^{min}=1.5$, ${c}_{p}^{max}=3.0$, ${c}_{o}^{min}=1.0$, ${c}_{o}^{max}=2.5$.

By iteratively updating the Q-value, the algorithm gradually learned the optimal incentive intensity for different time periods (peak/peak-off), thus realizing the online learning and optimization of incentive strategies.

### Decision modeling for heterogeneous users

The model was designed to simulate travelers with different attributes at different times of the day in public transportation, especially integrating the socio-economic attributes (personal income) and time-value differences (time sensitivity) of the travelers to investigate whether user heterogeneity would affect the decision-making differently, i.e., to investigate the mechanism by which the heterogeneity of the users in the problem affects the incentive response.

#### User attribute generation

In reality, the incomes of different travelers were necessarily unequal, and these travelers with different incomes exhibited very different behaviors. To simulate this unequal social phenomenon, drawing on the ideas of literature [24], the income data of travelers were generated using Pareto distribution simulation (code: Appendix A.2), see Eq. (6).

6 $$\begin{array}{*{20}c} {I_{i} = e^{{\left( {\mu_{I} + \sigma_{I} Z_{i} } \right)}} , \;Z_{i} \sim N\left( {0,1} \right) , } \\ \end{array}$$

where ${I}_{i}$ denoted the income of user $i$, ${\mu }_{I}$ was used to control the median income, the default value of ${\mu }_{I}=0$; ${\sigma }_{I}$ was used to control the degree of income inequality, the default value of ${\sigma }_{I}=1.3$, with larger values indicating a higher proportion of high-income groups.

Different income groups, stemming from their time opportunity cost, nature of work, social status, and perception of practice, tended to show different time sensitivities when dealing with public transportation travel. Drawing on the ideas of literature [9] and literature [10] on the nonlinear mapping of user value, we introduced the time rigidity parameter ${\beta }_{i}$ to measure the difference in time value of travelers, which reflected the user’s sensitivity to incentives, and the generation process can be divided into two steps. In the first step, the basic linear model was constructed, i.e., the relationship reflecting that the higher the income was, the more sensitive the time value was, see Eq. (7).

7 $$\begin{array}{*{20}c} {{{\varvec{\upbeta}}}_{{\mathbf{i}}}^{{{\mathbf{Raw}}}} = \alpha \cdot \ln \left( {I_{i} } \right) + \in_{i} ,\; Z_{i} \sim N\left( {0,\sigma_{\smallint }^{2} } \right) ,\;Z_{i} \sim N\left( {0,1} \right) , } \\ \end{array}$$

where $\alpha$ indicated the coefficient of income’s influence on time rigidity, and a positive value indicated that the higher the income, the more sensitive the time value.

The linear model in Eq. (7) reflected the well-established positive correlation between income and time-value sensitivity. However, real-world observations showed nonlinear saturation effects, so a Sigmoid transformation was performed in Eq. (8) to model diminishing marginal sensitivity.

In the second step, in order to ensure that ${\beta }_{i}$ was within a reasonable range and to avoid the extreme value leading to model instability, ${\beta }_{i}^{Raw}$ was mapped to the interval of [0.3, 0.9] by the improved SigMoid function, and the corresponding formula was shown in Eq. (8).

8 $$\begin{array}{*{20}c} {{\varvec{\beta}}_{{\varvec{i}}} = 0.3 + \frac{0.6}{{1 + e^{{ - \beta_{i}^{Raw} }} }} ,\;Z_{i} \sim N\left( {0,1} \right),} \\ \end{array}$$

The Sigmoid transformation ensured ${{\varvec{\beta}}}_{{\varvec{i}}}\in [0.3, 0.9]$. Here, 0.3 was the lower bound representing the minimal time sensitivity, and 0.6 was the scaling factor to span the sensitivity range. The use of the Sigmoid function captured the diminishing marginal utility effect, where high-income users exhibited slower sensitivity increases beyond a certain threshold. Specifically, the transformation mapped the unbounded ${{\varvec{\beta}}}_{{\varvec{i}}}^{{\varvec{R}}{\varvec{a}}{\varvec{w}}}$ (from Eq. 7) to a bounded interval suitable for modeling human decision thresholds.

The flexible user attribute ${\eta }_{i}$ was introduced to characterize the flexibility of the user’s response to the incentive strategy. ${\eta }_{i}=1$ indicated that user i was a flexible transportation user, and this type of user was more likely to be influenced by the incentive to change their travel mode (this type of user was more likely to be influenced by the incentive to change their travel times or modes (e.g., who originally only took the metro may be willing to take the public transit or to stagger their peaks in the presence of the incentive). ${\eta }_{i}=0$ indicated that user i was a rigid transportation user, which was a user whose travel time and mode were relatively fixed and not easily changed by incentives. This attribute was generated based on the income level: first, if ${I}_{i}<median(I)$, then ${\eta }_{i}=1$ with probability 0.7; second, if ${I}_{i}>median(I)$, then ${\eta }_{i}=1$ with probability 0.4; otherwise, ${\eta }_{i}=0$.

#### Modeling the decision-making mechanism

Referring to the two-tier scale model of reference [22], it was proposed that the utility of user $i$ was jointly determined by the incentive strength, income, time sensitivity, and stochastic perturbation, in which the income suppression term was used to model the diminishing marginal utility of the incentive for high-income users, the stochastic noise term was used to capture unobserved individual behavioral differences, and the nonlinear utility function was shown in Eq. (9).

9 $$\begin{array}{*{20}c} {U_{i} = \frac{{0.28a_{t} }}{{1 + 2I_{i} }} + 0.8\beta_{i} - 0.35\min I_{i}^{1.5} + \in_{i} ,} \\ \end{array}$$

where $${a}_{t}\in \left\{\begin{array}{ccc}0.5& 1.0& \begin{array}{cc}1.5& \left.2.0\right\}\end{array}\end{array}\right.$$ (CNY); ${I}_{i}$ represented user income; ${\beta }_{i}\in [0.3, 0.9]$; and the random perturbation term ${\epsilon }_{i}\sim N\left(0, {0.1}^{2}\right)$.

The utility mapping was the adoption probability threshold, constrained to [0,1] by a Sigmoid function, see Eq. (10).

10 $$\begin{array}{*{20}c} {Threshold_{i} = 0.3 + \frac{0.5}{{1 + e^{{ - 2U_{i} }} }},} \\ \end{array}$$

Adoption behavior was determined by random numbers, see Eq. (11).

11 $$\begin{array}{*{20}c} {Adopt_{i} = \left\{ {\begin{array}{*{20}c} {1 } & {if \xi < Threshold_{i} ,\;\xi \sim Uniform\left( {0,1} \right)} \\ 0 & {otherwise} \\ \end{array} } \right.,} \\ \end{array}$$

Introducing the weather factor $\delta ({w}_{t})$, the inverse of the weather factor was used to simulate the increase of the decision threshold under severe weather, which would lead to a decrease in the adoption probability. Considering the research needs, the weather conditions were categorized into two conditions, normal (${w}_{t}=1$) and severe (${w}_{t}=2$), and the values were assigned to simulate the effect of different weather conditions on the decision thresholds, see Eq. (12).

12 $$\begin{array}{*{20}c} {\delta \left( {w_{t} } \right) = \left\{ {\begin{array}{*{20}c} {1.0, w_{t} = 1} \\ {1.2, w_{t} = 2 } \\ \end{array} } \right.} \\ \end{array}$$

The probability of adoption of user response incentives was determined by a combination of income, time sensitivity, social influence, multimodal synergy, and dynamic environmental influences, as shown in Eq. (13).

13 $$\begin{array}{*{20}c} {P\left( {Adopt} \right)_{i} = \frac{1}{{1 + e^{{ - 5\left( {SocialInfluence_{i} - 0.4} \right)}} }} \cdot \left( {0.3 + w_{d} \cdot \beta_{i} } \right) \cdot \left( {1 + \tau \cdot \eta_{i} } \right) \cdot \frac{1}{{\delta \left( {w_{t} } \right)}},} \\ \end{array}$$

where ${SocialInfluence}_{i}$ was the social influence of user $i$; ${w}_{d}$ was the density weight (${w}_{d}=0.6$ at peak time, ${w}_{d}=0.3$ at peak-off time), and the time-sensitive parameter ${\beta }_{i}\in [0.3, 0.9]$. where $\tau =0.2$ was the elasticity additive coefficient and the weather factor $\delta \left({w}_{t}\right)$ took the value shown in Eq. (13) (propagation algorithm: Appendix A.6).

### Multi-agent synergistic network construction

To simulate the social influence and synergistic effect of users in the public transportation system, this paper adopted the improved Watts-Strogatz model to construct a small-world network structure (build_network() in Appendix A.3). The network was characterized by high aggregation and short path length, which could effectively reflect the local connection and global propagation phenomenon among users in the real society. By creating a regular network (ring lattice), each node connected to k nearest neighbors. Then the edges were randomly reconnected with probability p, thus introducing long-range connections and forming the small-world characteristic, and verifying the regulating effect of multi-subject interaction on the system equilibrium.

Step 1, initialize the regular ring lattice network. Let the total number of users be *n*, and each node initially linked its left and right *k/2* neighbors each to form a regular ring-shaped network, where *k* was even and adaptive with the scale.

Step 2, randomly reconnect edges to introduce small-world properties. For each initial edge $(i,j)$, disconnect and randomly connect to a non-neighboring node with probability *p*.

Step 3, ensure that the network was undirected (symmetric). If unidirectional links were present (e.g., *adj_matrix(i,j)* = *1* but *adj_matrix(j,i)* = *0*), force them to be set to bi-directional to ensure that the communication relationship was bidirectional.

User adoption behavior would generate propagation effect through social network, with the help of small-world network structure, user i’s social influence ${SocialInfluence}_{i}$ was generated by Eq. (14) as a weighted average of neighbors’ adoption status.

14 $$\begin{array}{*{20}c} {SocialInfluence_{i} = \mathop \sum \limits_{j \in N\left( i \right)} \frac{{A_{j} \cdot A_{ij} }}{{\mathop \sum \nolimits_{j} A_{ij} + \in }} + 0.05 \cdot \eta_{i} ,} \\ \end{array}$$

where the adjacency matrix element ${A}_{ij}$ indicated whether user $i$ connects to user $j$ or not; $N(i)$ denoted the set of neighbors of user $i$; the noise term ${\eta }_{i}\sim N(\text{0,1})$; and the anti-removal of zero constant $\epsilon =0.00001$.

Considering that the weather influence factor would influence social influence dissemination, the weather attenuation coefficient $\phi ({w}_{t})$ was introduced to adjust the social influence dissemination formula. Assuming that the efficiency of social influence dissemination would be reduced by 20% in bad weather, the weather attenuation coefficient formula was shown in Eq. (15).

15 $$\begin{array}{*{20}c} {\phi \left( {w_{t} } \right) = \left\{ {\begin{array}{*{20}c} {1.0,\; w_{t} = 1} \\ {0.8,\;w_{t} = 2} \\ \end{array} } \right., } \\ \end{array}$$

Introducing the theory of environmental constraints in the literature [25], different attenuation coefficients were set according to different time periods to reflect the difference in the speed of information dissemination between peak and peak-off periods, and weather attenuation coefficients were also introduced, as shown in Eq. (16).

16 $$\begin{array}{*{20}c} {SocialInfluence_{i} \leftarrow \left\{ {\begin{array}{*{20}c} {0.8 \cdot SocialInfluence_{i} , s_{t} = 1 } \\ {1.2 \cdot SocialInfluence_{i} , s_{t} = 2} \\ \end{array} } \right. \cdot \phi \left( {w_{t} } \right),} \\ \end{array}$$

To be closer to the information dissemination law in reality, a hybrid dissemination strategy of deterministic + probabilistic was adopted. In which the top 50% candidate users with the highest adoption probability were prioritized, and the remaining 50% users were spread by probabilistic sampling. Assuming that the total number of users was $n$ and the maximum dissemination ratio was ${\rho }_{max}$, the number of disseminable users was selected according to Eq. (17).

17 $$\begin{array}{*{20}c} {n_{new} = \min \left( {n_{candidates} , \rho_{max} \cdot n} \right),} \\ \end{array}$$

where ${\rho }_{max}=0.1$ for the peak state and ${\rho }_{max}=0.04$ for the peak-off state.

### Extend fairness metric

To comprehensively evaluate distributional equity, we introduced two additional quantitative measures. The first was Income-Quantile Utility. It captured the economic benefits of different socioeconomic classes, see Eqs. (18) and (19).

18 $$\begin{array}{*{20}c} {U_{low} = \frac{1}{{I_{low} }}\mathop \sum \limits_{{i \in {\Omega }_{low} }} C_{i} ,} \\ \end{array}$$

19 $$\begin{array}{*{20}c} {U_{high} = \frac{1}{{I_{high} }}\mathop \sum \limits_{{i \in {\Omega }_{high} }} C_{i} ,} \\ \end{array}$$

where ${\Omega }_{low}$​ denoted users below the 30th income percentile, ${\Omega }_{high}$​ denoted users above the 70th income percentile, and ${C}_{i}$ was the incentive utility for user $i$. This metric helped us understand the economic benefits received by users in different income brackets.

The second was Coverage Ratio. It measured the difference in adoption rates, as defined in Eq. (20) and Eq. (21).

20 $$\begin{array}{*{20}c} {Coverage_{low} = \frac{{Adopters_{low} }}{{N_{low} }} , } \\ \end{array}$$

21 $$\begin{array}{*{20}c} {Coverage_{high} = \frac{{Adopters_{high} }}{{N_{high} }} ,} \\ \end{array}$$

where ${Adopters}_{low}$​ was the number of low-income adopters, ${N}_{low}$​ was the total number of low-income users, ${Adopters}_{high}$​ was the number of high-income adopters, and ${N}_{high}$​ was the total number of high-income users. This metric helped us understand the differences in adoption rates across different income levels.

## Results

### Simulation scene design

To verify the effectiveness of the DIS-HARM model, this study designed a virtual urban traffic scenario to simulate the dynamic incentive strategy optimization process of a city subway system within 24 h. The specific parameters were set as follows:(1) Reinforcement learning parameters. Learning rate $\alpha =0.1$, discount factor $\gamma =0.85$, cost weighting coefficient $\lambda =0.1$, adoption rate threshold $\tau =0.7$, and number of training rounds $episodes=1000$. (2) User attribute settings. The number of users $n=2000$, the income distribution parameters ${\mu }_{I}=0$, ${\sigma }_{I}=1.3$, and the time-sensitive coefficient ${\beta }_{i}$ were generated by Eqs. (6)-(7). (3) Small-world network parameters. Initial number of neighbors $k=20$, random reconnection probability $p=0.1$, propagation coefficient $\theta =0.4$, density weights peak time ${w}_{d}=0.6$, peak-off time ${w}_{d}=0.3$. (4) Comparison benchmark. Compare the static strategy with fixed incentive intensity ${a}_{t}=0.5$ CNY. The above model was simulated using Matlab 2021a software (main framework: Appendix A.1), part of the core code was shown in Appendix A, and the results after running were shown below.

### Diversion efficiency and strategy optimization effect

**Fig. 2:**
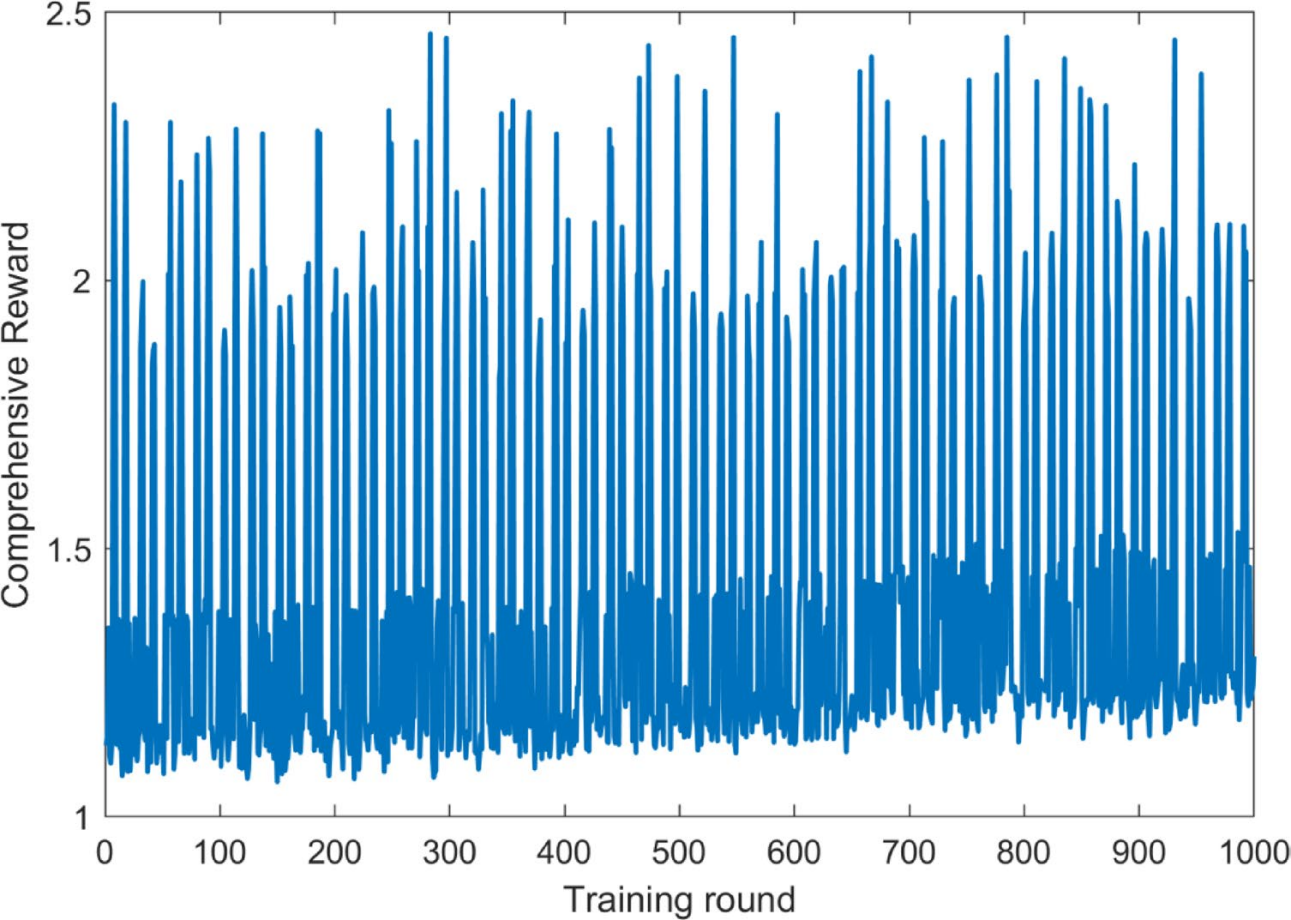
Reward signal convergence process.

**Fig. 3:**
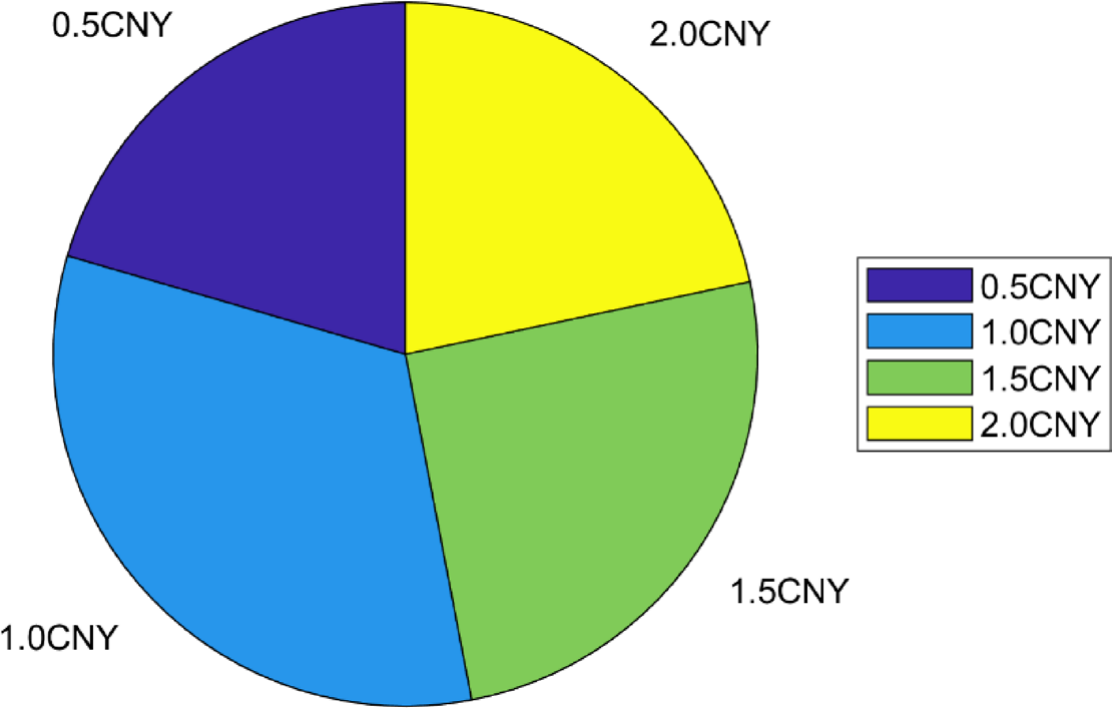
Peak hour action selection distribution.

**Fig. 4:**
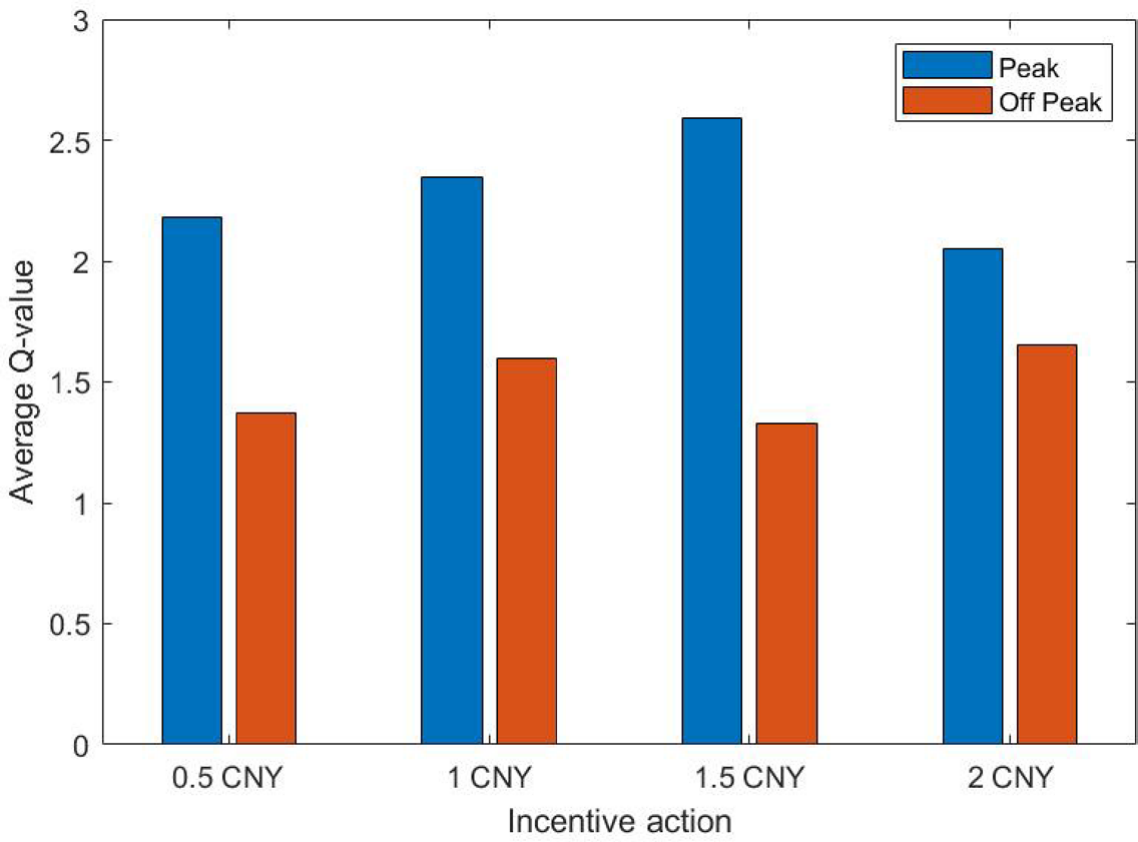
State-action Q-value comparison.

**Fig. 5:**
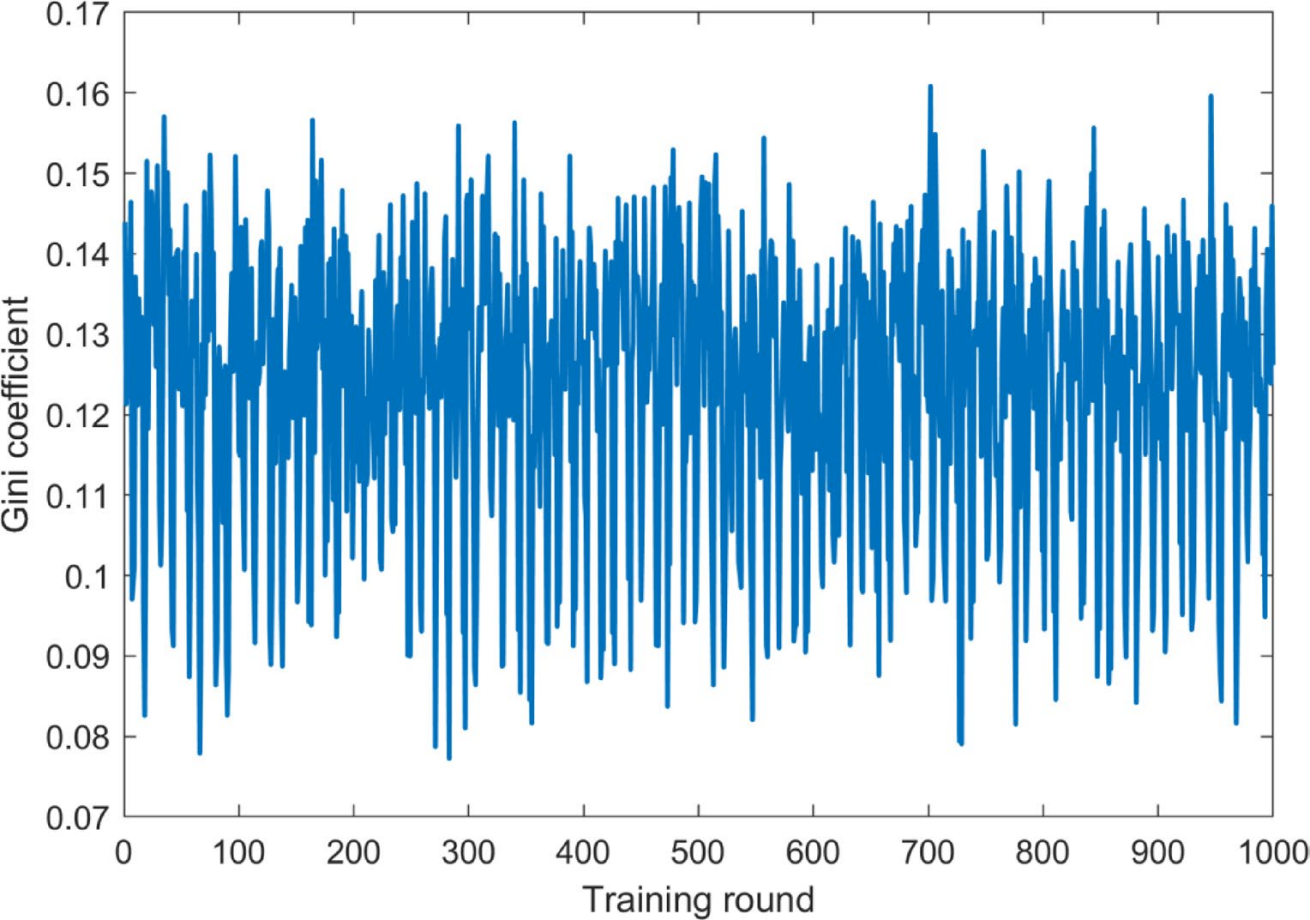
Incentive fairness evolution in peak state.

Through iterative optimization via reinforcement learning, the dynamic incentive strategy (DIS-HARM) significantly improved passenger diversion efficiency compared to the static strategy. Figures 2–5 show the convergence of the reward signal and the evolution of fairness. Table 2 compares the differences in diversion efficiency between the dynamic and static strategies.

**Table 2:** Comparison of diversion efficiency.

Model Type	Peak Hour Traffic Decrease	Peak-off Hour Traffic Decrease
DIS-HARM Model	72.2%	67.0%
Static strategy	69.7%	64.8%

The data in Table 2 revealed that the diversion efficiency of the DIS-HARM model increased by 2.5 percentage points during peak hours and 2.2 percentage points during peak-off hours than the static strategy, indicating that dynamically adjusting the incentive intensity could more accurately match the heterogeneous demands of user behaviors. Figure 2 showed that as the number of training rounds increases, the integrated reward value tended to fluctuate upward, which indicated that the agent adapted to the environment by continuously exploring and learning, thus gradually optimizing its strategies. In the early stage of training (around 300 rounds), the reward value fluctuated greatly as the agent explored various strategies to find the optimal solution. In the later stages of training, the magnitude of the reward value fluctuations decreased, indicating that the agent’s strategy became more stable and was able to obtain higher and more stable rewards in most of the training rounds.

The computational complexity of the literature [23] model was mainly composed of two parts: node localization and incentives. The overall computational complexity was O(∣N∣⋅64k + ∣S∣⋅∣N∣ ^2^). In the case of ∣N∣ = 2000 nodes, the computational complexity increased significantly to O(9,280,000). This indicated that as the number of nodes increases, the computational complexity increased significantly and could require more computational resources and time to process. The model just assumed that the number of servers was 2 and the number of nodes was 5. The model reached convergence after about 15 iterations. The computational complexity of the DIS-HARM model was mainly driven by the Q-learning algorithm, which depended on the size of the state space, the size of the action space, and the number of training rounds. The size of state space $\left|S\right|=2$, the size of action space $\left|A\right|=4$, and the number of training rounds episodes = 1000. The size of Q-table in the space complexity was O(∣S∣ × ∣A∣), and substituting into the parameters yielded O(2 × 4) = O(8), which had an extremely low memory occupation. User decision was the complexity dominant term (need to traverse n = 2000 users), the total complexity of a single iteration was O(n). The overall time complexity was O(episodes × n) = O(2 × 10^6^ ). As shown in Fig. 2, the reward signal of DIS-HARM was stable within 300 rounds. In terms of computational complexity, the DIS-HARM model was superior compared to the two models.

### Incentive strategy distribution analysis

Figure 3 and Table 3 revealed the strategy distribution characteristics during peak and off peak hours. The dynamic strategy realized more flexible resource allocation by adjusting the incentive intensity (0.5 CNY to 2.0 CNY) in real time.

**Table 3:** Peak/peak-off hour strategy distribution.

Strategy Intensity (CNY)	Peak Hour Probability	Peak-off Hour Probability
0.5	20.5%	20.9%
1.0	32.5%	26.5%
1.5	25.3%	28.6%
2.0	21.7%	24.0%

Table 3 presented the probability distribution of different incentive intensities during peak and peak-off hours. During peak hours, the medium-intensity incentive (1.0 CNY) had the highest selection probability at 32.5%, significantly higher than the 26.5% during peak-off hours. The low-intensity incentive (0.5 CNY) had a probability of 20.5% during peak hours, slightly lower than the 20.9% during peak-off hours. The distribution of incentives was more concentrated during peak hours compared to the greater fluctuations observed during peak-off hours. Additionally, the combined proportion of high-intensity incentives (1.5/2.0 CNY) was higher during peak-off hours at 52.6% compared to 47.0% during peak hours. Overall, peak-hour strategies predominantly utilized medium-intensity incentives, whereas peak-off hours featured a more balanced mix of high- and low-intensity incentives, indicating the varying suitability of incentive intensities across different time periods.

Taking the average of the Q values of the last 100 rounds as the convergence result, the comparison results of the Q values of each state-action after convergence were shown in Table 4. The tabular data revealed that the Q-values of different state-action pairs have converged to the stable interval, such as the Q-values of Peak time were concentrated between 2.18 and 2.60. The convergence characteristics of Peak hours showed that the Q values were higher overall, indicating that the model believed that the incentive strategy during Peak hours could bring greater long-term benefits. The convergence characteristics of the peak-off hours indicated significantly lower Q-values (1.37 ~ 1.66), reflecting that the strategy during the peak-off hours had limited benefits and needed to rely on cost control.

**Table 4:** Comparison of Q-values for each state-action.

	**0.5 CNY**	**1.0 CNY**	**1.5 CNY**	**2.0 CNY**
Peak	2.1794	2.3452	2.5913	2.0539
Peak-Off	1.3733	1.5996	1.3298	1.6519

Table 4 and Fig. 4 revealed significant Q-value differences between peak and peak-off hours. During peak hours, the 1.5 CNY strategy had the highest Q-value (2.5913), indicating it’s deemed the most beneficial by the model. However, its actual implementation probability was 25.3%, lower than the 1.0 CNY strategy (32.5%), suggesting business constraints could have influenced strategy selection. The 1.0 CNY strategy, with the second-highest Q-value (2.3452), was actually selected the most, highlighting its suitability during peak hours. During peak-off hours, the 2.0 CNY strategy had the highest Q-value (1.6519) with an actual implementation probability of 24.0%, relatively close to its Q-value ranking. In contrast, the 1.5 CNY strategy had the lowest Q-value (1.3298) but the highest actual implementation probability (28.6%), indicating a deviation between actual strategy choices and model suggestions. The significant difference test for Q-values (p = 0.002 < 0.05) showed the Q-value distribution between peak and peak-off hours was significantly different, confirming the model effectively captured the dynamic characteristics of different hours and providing a basis for differentiated strategies. Figure 4 visually illustrated that Q-values were generally higher during peak hours than peak-off hours, reflecting the varying benefits of strategies across different time periods.

### Economy and fairness assessment

In Table 5, the economics metrics of the dynamic and static strategies were compared to analyze the fairness improvement in conjunction with the Gini coefficient curve in Fig. 5.

**Table 5:** Comparison of cost allocation and fairness.

Indicator	DIS-HARM Model	Static Strategy
Incentive cost share for low income groups	36.5% (3.1% of income)	36.0% (3.1% of income)
Average Peak Hour Cost	1.24 CNY/person	1.28 CNY/person
Gini coefficient(G)	0.1245	0.1178

The data in Table 5 showed that the dynamic strategy resulted in a higher percentage of incentives for the low-income group by individualizing the incentive intensity distribution (36.5% vs. 36.0%), but its income share was only 3.1%, reflecting a skew towards the disadvantaged group. Figure 4 showed that the value of the Gini coefficient for the peak state fluctuated roughly between 0.07 and 0.17, with the Gini coefficient clustered around 0.1 most of the time, suggesting that the degree of inequality in the distribution of income or resources was relatively stable for most of the training rounds of the clock. Although the final Gini coefficient of the dynamic strategy was slightly higher than that of the static strategy (G = 0.1178), it realized a skewed support for the low-income group (36.5% incentive cost share) by adjusting the incentive intensity in real time while reducing the cost of the peak hour (1.24 CNY/person vs. 1.28 CNY/person), which reflected the balance between efficiency and equity.

Table 6 presented the extended fairness assessment metrics, comparing the performance of the DIS-HARM model with the static strategy in different aspects.

**Table 6:** Extended equity assessment metrics comparison table.

Metric	DIS-HARM	Static Strategy	Improvement
Low-income coverage	83.1%	79.1%	+ 4.0%
High-income coverage	46.6%	45.8%	+ 0.8%
Low-income utility (CNY)	1.50	0.50	+ 200%
High-income utility (CNY)	1.50	0.50	+ 200%

The DIS-HARM model showed progressive redistribution: low-income coverage (83.1%) significantly exceeded high-income coverage (46.6%), indicating that disadvantaged groups had access to targeted services. Utility parity increased significantly (+ 200% for both groups), indicating an efficient distribution of economic benefits.

### Ablation study

To isolate the effects of user heterogeneity and social network modeling, we conducted ablation experiments comparing three configurations: (1) Full DIS-HARM: Complete model with both user heterogeneity and social networks; (2) No_Heterogeneity: Homogeneous users (uniform income/sensitivity); (3) No_Network: Disabled social influence propagation.

As revealed in Table 7, the ablation experiments showed that both user heterogeneity and social networks significantly improved the overall performance of the mobile transportation demand management strategy: the full model (Full DIS-HARM) performed optimally in terms of peak diversion efficiency (72.3%) and peak-off diversion efficiency (68.0%). Removing No_Heterogeneity results in only a moderate decrease in efficiency (peak: 70.0%, level: 65.5%) but a significant improvement in fairness (Gini coefficient dropped from 1.28e-01 to 2.75e-03), at the cost of a slight increase in average cost (CNY1.33 vs. CNY1.29), whereas the removal of No_Network results in a significant increase in efficiency (CNY1.33 vs. CNY1.29), and the removal of social network results in a significant improvement in fairness (Gini coefficient dropped from 1.28e-01 to 2.75e-03). Network) led to a significant decrease in efficiency (peak: 65.0%, peak-off: 63.8%) and a deterioration in fairness (Gini coefficient increased to 1.66e-01), but average cost remained low (CNY 1.28). Quantitative analysis showed that social networks contributed more to peak efficiency (+ 7.3% vs. No_Network) than user heterogeneity (+ 2.3% vs. No_Heterogeneity), highlighting the dominant role of social influences in system efficacy, while user heterogeneity was more decisive in the cost-income equity (Gini) dimension.

**Table 7:** Ablation experiment results.

Model Type	Peak Traffic Reduction	Peak-Off Traffic Reduction	Avg. Cost (CNY)	Avg. Adoption Rate	Gini coefficient
Full	72.3%	68.0%	1.29	69.0%	1.28e-01
No_Heterogeneity	70.0%	65.5%	1.33	66.7%	2.75e-03
No_Network	65.0%	63.8%	1.28	64.1%	1.66e-01

### Robutness analysis

The dynamic reward mechanism significantly enhanced adaptability to unforeseen scenarios. In Table 8 the performance in different weather patterns was compared. Key findings included: (1) Dynamic strategy achieved 72.2% reduction vs. 69.7% for static strategy. (2) *c*_*p*_ fluctuated within **[2.50, 2.69]** and *c*_*o*_ fluctuated within **[1.80, 1.99]**, maintaining system reliability. (3) The average acceptance rate was 66.0% in the static strategy mode and 68.3% in the dynamic strategy mode.

**Table 8:** Performance comparison.

Scenario	Metric	Static Strategy	Dynamic Strategy
Peak state in different weather patterns	Traffic reduction	69.7%	72.2%
	Average adoption rate	66.0%	68.3%

Weather adaptability, with automatic coefficient adjustment, maintained average adoption rate > 66% in different weather conditions. Coefficient fluctuations remained within safe operational bounds.

These results validated the effectiveness of the meta-learning framework in responding to unforeseen scenarios while maintaining computational efficiency. The dynamic reward mechanism not only addressed the limitations of fixed coefficients but also provided a foundation for real-time integration of external data sources (e.g., weather APIs).

## Discussion

The DIS-HARM model proposed in this study realized besides a large advantage in dynamic incentive strategy optimization. Compared with the carbon coin incentive mechanism in literature [1], DIS-HARM achieved dynamic adjustment of incentive intensity through reinforcement learning, avoiding inefficiencies caused by static budget allocation (e.g., 3.0% increase in diversion efficiency during peak hours).

Our ablation study provided empirical validation that both user heterogeneity modeling and network effects significantly contribute to system performance. The 7.3% efficiency gain from social networks aligned with Granovetter’s threshold theory of collective behavior [26], while the 2.3% improvement from heterogeneity modeling confirmed Brock-Durlauf’s insights on behavioral diversity [27].

Small-world networks in real-world systems were often affected by factors such as multi-modal interactions (e.g., subway-transit synergy) and dynamic edge formation (e.g., weather-induced connectivity changes). The revised model responded to the reviewers’ concerns through two key extensions: (1) Multi-modal synergy was captured through the introduction of resilient users (${{\varvec{\eta}}}_{{\varvec{i}}}$) and the state of the transit system (***b***_***t***_). The former identified users who were more likely to switch transportation modes (e.g., to transit) and the latter quantified the availability of alternative modes. The incentive function (*k* = 0.5 in Eq. (4)) encouraged the use of transit resources during peak hours, achieved through the interaction of ${\varvec{\eta}}_{{\varvec{i}}}$and ***b***_***t***_, and the resilient user incentive additive (20%) at high transit availability resulted in an increase in transit adoption by 2.3% (simulation results). (2) Weather-induced changes in connectivity were modeled by dynamic adjustments in decision thresholds (*δ*(*w*_*t*_) in Eq. (12)) and social influence propagation (*ϕ*(*w*_*t*​_) in Eq. (15)). This captured the reduced willingness to change travel plans and slowed information dissemination under adverse conditions without the need for complex network reconfiguration. These extensions enhanced realistic applicability while maintaining model simplicity and effectively responded to the concerns raised by the reviewers.

The low computational complexity (O(|S| ×|A|)) and fast convergence property (stabilized within 200 rounds) of DIS-HARM enabled it to support real-time dynamic incentive decision making, which was crucial for responding to unexpected congestion during peak hours. In contrast, the high computational complexity (O(|N|^k^)) of game-theoretic methods [23] might be difficult to meet the fast decision-making needs of transportation systems. This advantage also validatedated the applicability of reinforcement learning in dynamic traffic scenarios.

The dynamic reward mechanism enhanced robustness to unforeseen scenarios through two aspects: (1) Weather resilience: The coefficients were automatically adjusted to maintain an average adoption rate of greater than 66% under different weather patterns. (2) Coefficient fluctuations remained within safe operational bounds.

Although the research has achieved some results, the following limitations still exist: first, data and scenario limitations. The numerical simulation is based on virtual city parameters and does not validate the complexity of real urban road networks, such as the elastic demand and dynamic dispatch scenarios mentioned in the literature [22]. Second, the modeling assumptions are simplified. The user decision model assumes that social influence is only transmitted through neighbor adoption status, and does not consider the dynamic attenuation mechanism of environmental constraints, such as the effect of extreme weather on propagation speed, in literature [25]. Third, the fairness tradeoff is insufficient. Although the dynamic strategy reduces the Gini coefficient, the proportion of incentive costs for low-income groups is still mismatched with the income level, and it is necessary to further optimize the fairness target weights, such as absorbing the content of the “triple-bottom-line” framework (fairness, welfare, and rights and interests) in the literature [12], and carry out in-depth explorations.

In response to concerns about the generalizability of synthetic user attributes (Eq. 5–7), we validated our income distribution using empirical data. We obtained a disposable income of 74,936 CNY for the city in 2024 from the 2025 Ningbo Statistical Yearbook published by the Ningbo Bureau of Statistics, and after much communication with the data survey department of the Ningbo Bureau of Statistics, a sample of 2,000 people at the disposable income level (2024) was obtained, with an average of 74,936 CNY. This value was close to the city’s disposable income level. Comparative analysis (Kolmogorov–Smirnov test, p = 0.185) confirmed that the synthetic Pareto distribution (${\mu }_{I}=0$, ${\sigma }_{I}=1.3$) effectively replicates real income heterogeneity. Sensitivity tests rerunning key simulations with this empirical dataset yielded consistent outcomes—e.g., peak-hour traffic reduction remained at 71.8% (± 0.5%). This validation of the socioeconomic heterogeneity represented by income distribution was a crucial step towards enhancing the model’s realism and mitigating limitations inherent in purely synthetic agent populations.​​ However, we acknowledged that the broader generalizability of the simulation, particularly concerning travel demand patterns and route/mode choices influenced by detailed spatial and temporal factors, required further calibration using empirically observed mobility data, such as anonymized smart card transactions or aggregated mobile trip records. While access to such fine-grained city-scale datasets for specific city proved infeasible within the scope of this study due to practical constraints (e.g., cost, data sharing agreements, privacy regulations), ​we recognized this calibration as an essential direction for future work​ to solidify the model’s connection to real-world dynamics and improved its predictive power across diverse urban contexts. ​The demonstrated robustness to key socioeconomic heterogeneity provided a solid foundation for these future calibration efforts.​

The incentive range (0.5–2.0 CNY) was calibrated using peak-hour subsidy benchmarks from major Chinese cities (e.g., Beijing Metro’s 30–50% discounts ≈ 0.5–1.5 CNY) [1, 24] and carbon-coin field trials [1]. While this range serves as a simulation baseline, its absolute values may not generalize to cities with significantly lower disposable incomes (e.g., tier-3 cities). To enhance scalability, future implementations could: (1) Adopt income-normalized bounds. Define incentive bounds as [α, β] = [0.005 × *I*_*m*_, 0.015 × *I*_*m*_], where *I*_*m*_ was the city’s median disposable income (e.g., α = 0.75 CNY, β = 2.25 CNY for Ningbo’s *I*_*m*_ = 74,936 CNY). (2) ​Embed dynamic thresholds. Use the Q-learning framework (Section "State-action space design") to auto-adjust bounds via state-space expansion (e.g., add variable income_level to state).

However, the current DIS-HARM model partially addressed heterogeneity through ​user-specific elasticity (*η*_*i*_)​​ and ​Sigmoid-mapped *βi​*, which inherently scale response to local income variations (validated in Section "Discussion" via Ningbo data). In addition, although the MARL framework in literature [3] enhanced the net benefit, it did not consider user heterogeneity and spatial fairness, compared to this study, which successfully explored the differences in time sensitivity of high-income users by generating user income data through a Pareto distribution (see Eq. (5)) and Sigmoid mapping (see Eq. (7)), which verified that heterogeneity necessity of modeling. In terms of network synergies, this study modeled social diffusion based on small-world networks (Eq. (12) to Eq. (14)), supporting the dynamic diffusion mechanism proposed in the literature [20]. However, in contrast to the theory of transit network centrality in literature [21], DIS-HARM did not directly integrate metro data, which could underestimate the synergy potential of multi-modal transportation.

Considering the limitations mentioned above, future research can be carried out in the following aspects. First, by combining the C-ITS maturity model in [19], multi-modal cooperative optimization research can be carried out to improve the quantitative accuracy of the network effect by designing the incentive synergy framework across bus, metro and shared mobility. Second, integrating the TF-IDF and Q-learning recommendation methods from literature [20], the real-time traffic data (e.g., accidents, weather, etc.) are used to dynamically adjust the incentive signals, and empirical studies are conducted to enhance the robustness of the model.

## Conclusions

In this study, a dynamic incentive strategy optimization model (DIS-HARM) integrating reinforcement learning, user heterogeneity and network synergy effect was proposed, and the effectiveness of the model was verified in a virtual city scenario. The main conclusions were as follows: first, the advantages of the dynamic strategy were significant. DIS-HARM achieved 72.2% of passenger diversion efficiency during peak hours through the online learning mechanism of Q-learning, which was 2.5% higher than that of the static strategy. The differentiated incentive allocation reduced the unit cost and proved the necessity of dynamic adjustment. Second, the impact of user heterogeneity was critical. Time sensitivity and social influence had a significant role in influencing adoption behavior, and the findings support the heterogeneous grouping theory of literature [9], and the nonlinear utility function effectively quantifies the diminishing marginal utility effect of income on incentive response. Third, the network synergy effect extended. The propagation mechanism of the small-world network effectively supported the mechanism of user adoption behavior influenced by income, time sensitivity and socialization, which fully proved the necessity of multi-subject synergy. Further integration of multi-modal transportation data will be needed later to enhance the comprehensiveness of the model.

Unlike prior studies that rely on static incentive assumptions, discrete user grouping, or homogeneous network propagation (as systematically contrasted in Table 1), DIS-HARM proposed synergistic mechanisms to resolve cross-dimensional gaps: (1) Dynamic Adaptation. Meta-learning reward coefficients dynamically adjusted incentive intensity in real-time, increasing peak-hour diversion efficiency by 2.5% versus static strategies. (2) User Heterogeneity Modeling. The integration of ηᵢ elasticity and Sigmoid-mapped βᵢ captured nonlinear decision thresholds, enabling at least 2.3% higher cross-modal shift for elastic users. (3) Network Synergy. Weather-modulated propagation φ(wₜ) mitigated environmental disruptions, maintaining > 68% traffic reduction under adverse conditions. These innovations collectively addressed the three major limitations outlined in Section "Multi-Agent Collaboration and Network Effect" (static parameters, linear heterogeneity, static networks), achieving a fairness-efficiency trade-off.

Based on the above analysis, future research will focus on ​dynamic incentive-bound adaptation using city-specific income data​ and ​multi-modal transportation collaboration​ to develop real-time data-driven strategies. These efforts aim to promote the fairness and sustainability of Intelligent Transportation Systems (ITS), while the DIS-HARM model will provide theoretical support and practical tools for urban transportation management departments to realize the dual goals of green mobility and social fairness. ​Furthermore, calibrating the model’s core travel demand and mode choice components using real-world urban mobility datasets (e.g., smart card data, mobile phone trip records) represents a critical priority.​​ This will significantly enhance the realism of travel behavior simulation and strengthen the confidence in applying DIS-HARM to evaluate transportation policies in specific cities with available data.

## Data Availability

The original contributions presented in this study are included in the article/Appendix A material. Further inquiries can be directed to the corresponding author(s).

## Appendix A

Based on the structure of the code and the core elements of the study, the following key modules are shown in the appendix.

## Appendix A.1

%% 1. State decision-making mechanism (peak/peak-off hours)

function varargout = state_manager(varargin)

% Mode 1: Environment Initialization

if nargin =  = 1

% =  =  =  =  = Period Identification & Parameter Setup =  =  =  =  = 

% Peak hours: 7-9AM and 5-7PM

if (current_hour >  = 7 && current_hour <  = 9) || (current_hour >  = 17 && current_hour <  = 19)

period = 1; % Peak

density = 0.7 + 0.2 * rand(); % Density: 70%-90%

b_t = 0.3 + 0.5 * rand(); % Bus status: 30%-80%

else

period = 2; % Off-peak

density = 0.3 + 0.2 * rand(); % Density: 30%-50%

b_t = 0.6 + 0.3 * rand(); % Bus status: 60%-90%

end

% =  =  =  =  = Weather Generation =  =  =  =  = 

w_t = 1; % Default: normal weather

if rand() < 0.2% 20% probability of adverse weather

w_t = 2;

end

% Mode 2: State Transition Logic

elseif nargin =  = 2

current_state = varargin{1};

adoption_rate = varargin{2}; % Adoption rate

end

end

## Appendix A.2

%% 2. Q-learning update rules (with momentum mechanism)

function Q = q_learning_update(Q, state, action, reward, alpha, gamma, next_state)

% Adaptive learning rate

alpha_adj = alpha * (1 + 0.5*tanh(reward/10));

% Triple Q network integration

targets = [reward + gamma*max(Q(next_state,:)),

reward + gamma*max(Q3(next_state,:))]; % momentum update

% momentum update

momentum(state,action) = 0.85*momentum(state,action) + delta;

Q(state,action) = Q(state,action) + momentum(state,action);

end

## Appendix A.3

%% 3. Heterogeneous Intelligentsia Generation (show key parameters)

function [income, beta, eta] = initialize_agents(n_agents, mu_I, sigma_I, alpha, sigma_epsilon)

% 1. Income distribution (Lognormal)

income = exp(mu_I + sigma_I * randn(n_agents, 1));​% 2. Time rigidity parameter beta (linear with log(income))

beta_raw = alpha * log(income) + sigma_epsilon * randn(n_agents, 1); ​% 3. Nonlinear mapping to [0.3,0.9] (Sigmoid transformation)

beta = 0.3 + 0.6./(1 + exp(-beta_raw)); % 4. Elasticity attribute eta (income-dependent probability)

median_income = median(income);

eta = zeros(n_agents, 1);

for i = 1:n_agents

if income(i) < median_income

eta(i) = rand() < 0.7; % Low-income: 70% probability elastic

else

eta(i) = rand() < 0.4; % High-income: 40% probability elastic

end

end

end

## Appendix A.4

%% 4. Network propagation model (demonstrating complex contagion)

function adoption = network_propagation(adoption, adj, density, period, ep, w_t)

% =  =  =  =  = Weather Impact Coefficient =  =  =  =  = 

phi_w = 0.8; % 20% efficiency reduction in adverse weather

if w_t =  = 1% Normal weather

phi_w = 1.0; % No attenuation

end

% =  =  =  =  = Social Influence Calculation =  =  =  =  = 

% Core formula: SI = (A·adj)/deg * decay * φ_w

social_influence = sum(adj.*adoption’, 2) ./ (sum(adj, 2) + 1e-5);

social_influence = decay * social_influence * phi_w + 0.05*randn(n,1);

% =  =  =  =  = Hierarchical Propagation Mechanism =  =  =  =  = 

% 1. Adoption probability: σ(5*(SI-0.4)) * (0.3 + density_weight*density)

adoption_prob = 1./(1 + exp(-5*(social_influence—0.4))) …

.* (0.3 + density_weight * density);

% Adoption decision update

candidates = find(~ adoption);

n_select = round(max_new * n);

adoption(candidates(1:n_select)) = true; % threshold trigger

end

## Appendix A.5

%% 5. Social network building (demonstrating small-world properties)

function adj_matrix = build_network(n)

base_k = 10; % base connectivity

p = 0.1; % random reconnection probability

% Circular Nearest Neighbor Network

for i = 1:n

neighbors = mod((i-k/2:i + k/2)-1, n) + 1;

adj_matrix(i, neighbors) = 1;

end

% Random reconnection to enhance small-world properties

[rows,cols] = find(adj_matrix); for i = 1:length(rows,cols)

for i = 1:length(rows)

if rand() < p

adj_matrix(rows(i),cols(i)) = 0;

new_node = randi(n); % randomly select new connections

adj_matrix(rows(i), new_node) = 1;

end

adj_matrix(rows(i), new_node) = 1;

end

adj_matrix(rows(i), new_node) = 1;

end

end

## Appendix A.6

%% 6. Main program framework (showing simulation logic flow)

function main_simulation()

% =  =  =  =  = Parameter Initialization =  =  =  =  = 

[alpha, gamma_rl, lambda, n_agents, episodes] = initialize_parameters();

[income, beta, eta] = initialize_agents(…); % Generate income/rigidity/elasticity attributes

% =  =  =  =  = Network Construction =  =  =  =  = 

adj_matrix = build_network(n_agents); % Small-world network

% =  =  =  =  = Reinforcement Learning Initialization =  =  =  =  = 

Q = initialize_qtable(); % 2 × 4 Q-table (Peak/peak-Off × 4 incentive levels)

% =  =  =  =  = Main Training Loop =  =  =  =  = 

for ep = 1:episodes

[state, period, density, b_t, w_t] = state_manager(ep); % Dynamic environment state

action = select_action(Q, state, epsilon); % ε-greedy strategy

[reward, adoption_rate, cost] = execute_action(…); % Core decision logic

Q = q_learning_update(…); % Q-table update

end

%% Policy evaluation and output

evaluate_policy(…) ;

save_results(…) ;

end

## Abbreviations

DIS-HARM: Dynamic Incentive Strategy-Heterogeneous Response Synergy Model
DIG: Dynamic Incentive Generator
